# 

**Published:** 1896-08

**Authors:** 


					﻿Vol. XVI.
AUGUST, 1896.
NO. 8?
THE
HOMEOPATHIC pHY^lClAfl,
A Monthly Journal of Homœopathic Materia Medica
and Clinical Medicine. •
“ He it a freeman whom truth makes free, and all are slaves beside.”
u Seek the Truth:-come whence it may, cost what it will.”
EDITED AMD PUBLISHED BY
WALTER M. JAMES, M. D.
PHILADELPHIA:
USTo. 1231 LOCUST STtdIKIZET.
LONDON!'
ALFRED HEATH & CO., Sole Agents for Great Britain,
114 Ebury Street, Eaton Square.
rYearly Subscription, $2.50, invariably in advance. Sinyle numbers, 95 els.
Entered- at the Philadelphia Poit-Offioe aa Becond-Claii Mall Matter.
				

